# Achievements, Challenges and Promises of Minimally Invasive Liver Transplantation

**DOI:** 10.3389/ti.2026.15366

**Published:** 2026-02-25

**Authors:** Clara Gomez, Ismail Labgaa, Elias Karam, Federica Dondero, Nassiba Beghdadi, Christian Hobeika, Safi Dokmak, Mickaël Lesurtel

**Affiliations:** 1 Department of HPB Surgery and Liver Transplantation, APHP-Nord, Beaujon Hospital, Département médico-universitaire (DMU) DIGEST, Clichy, France; 2 Université Paris Cité, Paris, France; 3 Department of Visceral Surgery, Lausanne University Hospital (CHUV), Lausanne, Switzerland; 4 Faculty of Biology and Medicine (FBM), University of Lausanne (UNIL), Lausanne, Switzerland; 5 Institut national de la recherche et la santé médicale (Inserm) UMR1327 ISCHEMIA Membrane Signaling and Inflammation in reperfusion injuries, Université de Tours, Tours, France; 6 Institut national de la recherche et la santé médicale (Inserm), UMR-S1149, Centre de Recherche sur l’Inflammation (CRI), Université Paris Cité, Paris, France

**Keywords:** laparoscopy, minimally invasive surgery, minimally invasive liver transplantation, robotic surgery, liver transplantation

## Abstract

The integration of minimal invasive (MIS) techniques in liver transplantation (LT) emerged as a natural progression following advances in laparoscopic and robotic hepato-pancreato-biliary surgery. However, it poses specific challenges that are inherent to LT. Chronologically, it is a recent topic that only emerged 2 decades ago in donors and recently in recipients, but it has showed a meteoric rise with tremendous progress over the last years. This review aimed to provide a comprehensive yet synthetic overview of the available data on minimal invasive liver transplantation (MILT), for both donor hepatectomy (DH), recipient hepatectomy and graft implantation. Developments were numerous: top-notch technical skills have not only been reported but have foremost been performed worldwide by an increasing number of groups. Technology also played a central role, as exemplified by the integration of 3D visualization techniques, the utilization of indocyanine green (ICG) near-infrared fluorescence camera system or the use of robotic technology. Research efforts finally illustrated this progress with a rapid rise of number of publications and adoption. The present analysis of the available data permitted to identify gaps that may be valuable to explore by future research projects.

## Introduction

Liver transplantation (LT) is the best therapeutical option for a wide range of end-stage liver diseases, acute liver failure, and some liver malignancies. LT has been increasingly performed with approximately 41,000 procedures worldwide in 2023 [1].

Over the past decades, hepatic minimally invasive surgery (MIS) has been developed, with both laparoscopic and robotic approaches [2, 3]. The main reported benefits of these techniques include reduced bleeding, a lower inflammatory response to trauma, decreased postoperative pain, improved cosmetic outcomes, and faster postoperative recovery [4]. The first laparoscopic liver resection was reported by H. Reich in 1991 [5]. Since then, MIS indications have expanded to include increasingly complex procedures. The first laparoscopic left lateral sectionectomy (LLS - segments II and III) in a living donor was reported by Cherqui et al. in [6] and 10 years later the first laparoscopic living donor right hepatectomy was described by Soubrane et al. [7]. These techniques then spread to Asia (South Korea) in particular where living donor liver transplantation (LDLT) is much more developed, and since 2016 attention has shifted toward the robotic approach [8]. However, partial liver resection from a living donor has been controversial, as it exposes a healthy individual to surgical morbidity and mortality and may impact long-term quality of life. Recent studies have shown that laparoscopic donor hepatectomy (L-DH) is feasible and safe when performed in an experienced liver transplant centre on selected donors [9–11].

Even though MIS was developed in living donors, it was only later applied to recipients. In 2011 Eguchi et al. described a hand assisted laparoscopic approach using MIS for liver mobilization, but a short midline incision was required for the subsequent explantation and implantation [12]. In 2019, the first laparoscopic total explant hepatectomy was reported by Dokmak et al. at Beaujon Hospital in France [13].

Although MIS in LT only implicates highly specialized hospital centers, it is considered a significant LT breakthrough. The present article aims to provide a thorough but synthetic overview of minimally invasive liver transplantation (MILT) and its different subdomains. It first focuses on the different aspects of the procedures and results in the donor, followed by a state-of-the art in the recipient.

## Materials and Methods

A detailed description of the methods is available in Supplementary Methods.

## Results

### Minimally Invasive Liver Transplantation at a Glance

**FIGURE 1:**
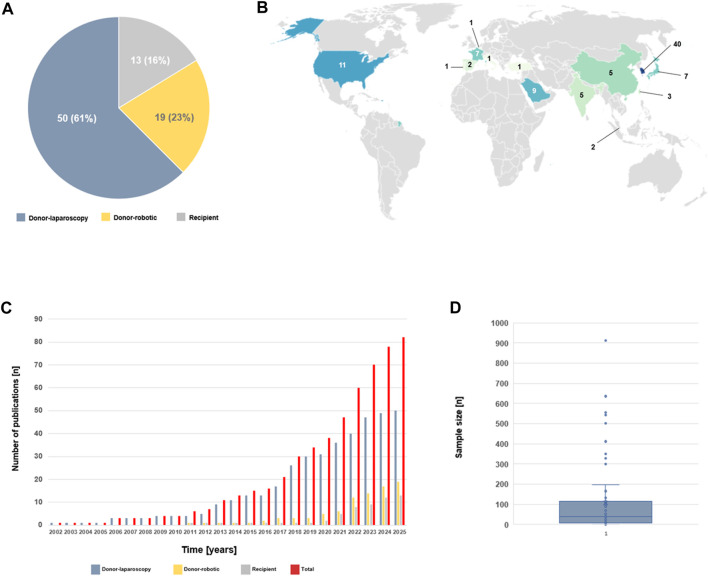
Minimally Invasive Liver Transplantation. Overview of the available studies on MILT (n = 82). **(A)** Pie chart illustrating the distribution between laparoscopic donor hepatectomy (L-DH), robotic DH (R-DH) and minimal invasive techniques in the recipients. **(B)** Global map illustrating the number of publications per country. **(C)** Bar plots showing the chronological evolution of publications in the field. **(D)** Box plot illustrating sample size of the available studies.

Our review of the literature identified a total of 82 publications on MILT [6–8], [10–88]. Most of them (50 studies, 61%) reported laparoscopic donor hepatectomy (L-DH) whereas reports on robotic donor hepatectomy (R-DH) and MIS techniques in recipients represented 19 (23%) and 13 (16%) articles, respectively (Figure 1A). In term of scientific contributions, Republic of South Korea (40 contributions), United States of America (11 contributions), Saudi Arabia (9 contributions), Japan and France (7 contributions, each) appeared as the leading countries (Figure 1B). While the first report on MILT was published in 2002, the number of publications remained relatively constant during the following decade and started rising upon 2017 (Figure 1C). Likewise, the purport of these articles has progressively increased, partly illustrated by larger sample sizes over the years (Figure 1D).

### Minimal Invasive Donor Hepatectomy (MIDH)

#### Laparoscopic Donor Hepatectomy (L-DH)

Laparoscopic donor hepatectomy (L-DH) was first reported in 2002, performed in two young parents in whom a left lateral sectionectomy (LLS) was performed and transplanted to their 1-year old sons suffering from biliary atresia [6]. Both donors and recipients recovered uneventfully and liver grafts showed excellent function. A decade later, striking progress were achieved to develop L-DH in pediatrics and adults, in particular in Asian countries such as Republic of South Korea. Literature on L-DH entails >50 peer-reviewed articles, detailed in Supplementary Table S1.

##### Overview of Laparoscopic Donor Hepatectomy (L-DH) Results

**FIGURE 2:**
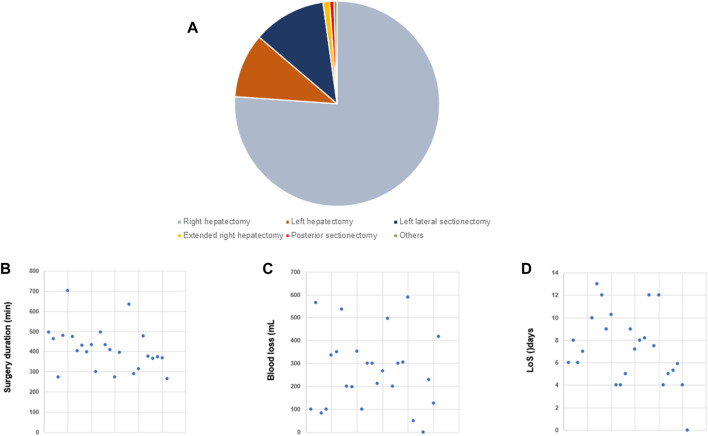
Laparoscopic donor hepatectomy (L-DH) Overview of selected studies on L-DH (n = 26). **(A)** Pie chart illustrating the distribution between the different types of partial hepatectomy. **(B)** Dot plot of surgery duration [minutes]. **(C)** Dot plot of blood loss [mL]. **(D)** Dot plot of length of stay (LoS) [days].

Twenty-six studies were selected for analysis [7, 11, 15, 19, 20, 22–24, 28, 31, 38, 42, 56, 60, 61, 64, 66, 68, 69, 71, 74, 77–79, 81, 82], yielding a total of 2404 patients. Most studies reported experiences of pure L-DH whereas a hybrid approach was also used. Right hepatectomy (RH) represented most procedures (Figure 2A). Conversion was requested in 30 patients (1.3%) (Table 1). Duration of surgery averaged 400 min (Figure 2B) and blood loss ranged from 100–600 mL (Figure 2C). No case of mortality was reported but 266/2404 (11.1%) and 95 (4%) patients developed overall and severe complications, respectively (Table 1). Most patients stayed 6–10 days at hospital after surgery (Figure 2D). Overall, these results demonstrate safety of L-DH.

**TABLE 1:** Conversion rates and incidence of adverse events in minimally invasive liver transplantation in the 82 listed studies.

​	Conversion	Overall complications	Major complications	Mortality
Laparoscopic DH	30/2404 (1.3%)	266/2404 (11.1%)	95/2404 (4%)	0/2404
Robotic DH	22/1629 (1.4%)	145/1629 (8.9%)	38/1629 (2.3%)	0/1629
Recipient	10/39 (25.6%)	5/39 (12.8%)	2/39 (5.1%)	1/39 (2.6%)
MILT	62/4072 (1.5%)	416/4072 (10.2%)	135/4072 (3.3%)	1/4072 (0.02%)

These data provide an overview on the outcomes of patients undergoing L-DH but it must obviously be stratified for each specific procedures (e.g., RH vs. LLS). Unfortunately, data comparing outcomes after RH, left hepatectomy (LH) and LLS are lacking, because most studies reported series of a specific procedure for which the authors gained sufficient experience. Rare studies included different procedures; although outcomes were excellent for each specific procedures, data reasonably showed a trend toward higher complications rates after RH as opposed to LH or LLS [48].

The added value of L-DH on cosmetic and patients’ satisfaction was also reported by several studies, as opposed to open donor hepatectomy (O-DH) [77, 82].

##### Patients’ Selection and Predictors of Adverse Outcomes

Although patients’ selection is paramount, most studies did not detail their selection criteria and/or did not precise whether specific conditions should be considered as contraindications for L-DH. Of note, the selection criteria of certain groups varied overtime, as exemplified by two groups that excluded donors with vascular or biliary anatomical variants in the initial phase of their experience with RH L-DH but thereafter extended their criteria and also included patients with anatomical variants [53, 63].

Important efforts were pursued to conduct research to assess safety and eventual benefits of L-DH. As an example, Rhu et al. thoroughly analyzed a monocentric cohort of 636 donors undergoing L-DH in South Korea [11]. Not only providing classical endpoints such as overall/major complications, mortality, and biliary complications, they also assessed postoperative bleeding, reoperation, and readmission rates that reached 6%, 2.2% and 5.2%, respectively. Furthermore, they identified risk factors of specific types of complications in donors: the presence of 2 hepatic arteries was associated with an increased risk of biliary leakage, whilst the Pringle maneuver appeared to be protective against this complication. Similarly, a multicentric Korean study including 543 patients aimed to identify factors associated with adverse events in to predict safety and thereby to facilitate patient selection [29]. BMI >30 kg/m2 was a predictor of higher conversion rate whereas graft weight >700 g and surgery duration >400 min predicted higher risk of overall- and major complications. In a recent study comparing L-DH and O-DH, multiple portal veins were identified as an independent predictor of major- (OR, 5.75; 95% CI, 1.28-25.79; p = 0.022) and biliary (OR, 3.84; 95% CI, 1.71-8.69; p = 0.001) complications, in donors [15].

##### Comparison With Open Approach

Subsequently, authors naturally aimed to determine whether L-DH was comparable or superior to O-DH. A cohort study reviewed 894 donors and conducted propensity score matching (PSM) for a head-to-head comparison of 198 donor-recipient pairs [42]. No case of mortality was observed. Compared to O-DH, L-DH was associated with longer duration of surgery (290 vs. 271 min, p < 0.001), longer time to remove the liver from the abdomen (211 vs. 166 min, p < 0.001) and longer warm ischemia time (12 vs. 4 min, p < 0.001), but reduced length of stay (LoS) (8 vs. 9 days, p < 0.001) and comparable overall complication rates (6.1% vs. 10.6%, p = 0.102); no difference in recipient survival was highlighted (p = 0.935). Another recent study also used PSM to compared both laparoscopic (n = 329) and open (n = 3019) approaches in living donors, and showed similar results [15].

##### Outcomes After Laparoscopic Donor Hepatectomy (L-DH)

Reporting their initial experience on L-DH in a cohort of 54 patients, Kwon et al. also analyzed recipients’ outcomes [63]: biliary and arterial complications occurred in 31.5% and 2.7%, respectively whereas graft failure was reported in 5 (9.3%) patients. A PSM analysis comparing L-DH and O-DH in 220 pediatric transplantations showed similar outcomes for recipients [67]. Park et al. also conducted a PSM analysis comparing 72 recipients from O-DH and L-DH, showing no difference for major complications (40.3% vs. 47.2%, p = 0.397), graft failure (4.2% vs. 5.6%, p = 0.699) and mortality (2.8% vs. 4.2%, p = 0.657) [57].

Cho et al. compared outcomes in both donors and recipients after laparoscopic RH *versus* laparoscopic right posterior sectionectomy [32]. Overall outcomes for recipients showed major complications and mortality rates of 36.5% and 2.3%, respectively, and comparison further detected higher rates of major complications after laparoscopic right posterior sectionectomy as opposed to laparoscopic RH (62.5% versus 35.2%, p = 0.034). Kim et al. identified multiple bile ducts as a predictor of bile leakage and biliary stricture in the recipients [15].

##### Technical Considerations

One may reasonably question the feasibility of implementing L-DH, particularly in Western countries. Encouraging data demonstrated the feasibility to develop programs dedicated to L-DH in Western countries with good outcomes [10, 31]. This raises the question of the learning curve, unfortunately barely investigated. Cumulative sum method (CUSUM) of the operative time of a single surgeon who performed 100 L-RH, showed a continuous fall after 43 operations, which was used as a cut-off to split the retrospective cohort in two groups (i.e., initial n = 43, and recent n = 57) [49]. In comparison to the initial group, surgery duration (282 vs. 181 min, p < 0.01) and length of stay (7.1 vs. 5.8 days, p < 0.01) were shorter in the recent group while overall complications rate was comparable (1.8% vs. 9.3%, p = 0.1). Following a similar approach, another group established that 1 year including 115 patients was sufficient to standardize the procedure [62].

Visualization techniques is also an important point. Although data comparing 2D versus 3D technologies are not yet available, recent studies mostly used 3D techniques. As an example, Kwon et al. reported switching from 2D to 3D during the study period [63], and rapidly recognized the advantages offered by 3D vision.

Likewise, indocyanine green (ICG) near-infrared fluorescence camera system has gained important interest and is more and more often utilized to facilitate the visualization of bile duct division and/or to demarcate the exact midplane [42]. As energy-sealing devices are more likely to be used in MIS, and they are presumably at higher risk of causing thermal damages to the microvasculature surrounding bile ducts. Offering the option to accurately delineate the biliary tree before transection, ICG may be particularly valuable to prevent biliary injuries.

#### Robotic Donor Hepatectomy (R-DH)

Robotic donor hepatectomy (R-DH) remains restraint to the experience of a small number of centers and surgeons that have developed the specific skills and expertise. Consequently, reports on the topic are scant, with only 20 publications [8, 14, 16–18, 20, 25, 26, 34, 36, 40, 41, 44, 46, 54, 55, 70, 80, 87, 88] retrieved from the literature (Supplementary Table S2). Those included 3 case reports, 2 case series, 11 cohort studies and 4 case-match studies. Five and 6 studies were conducted in South Korea and Saudi Arabia, respectively. Median sample size was 64 (12-116), heterogeneously varying from 1 to 913 patients.

In 2011, Giulianotti et al. reported the first case of robotic right hepatectomy for LDLT [80]. The procedure was exclusively performed with a minimal invasive technique and the specimen was extracted through a small lower midline incision. Cold and warm ischemia were limited to 25 and 35 min, respectively, and both the donor and the recipient showed an uneventful postoperative course. Subsequently, publications on the topic showed a meteoric rise.

##### Overview of Robotic Donor Hepatectomy (R-DH) Results

**FIGURE 3:**
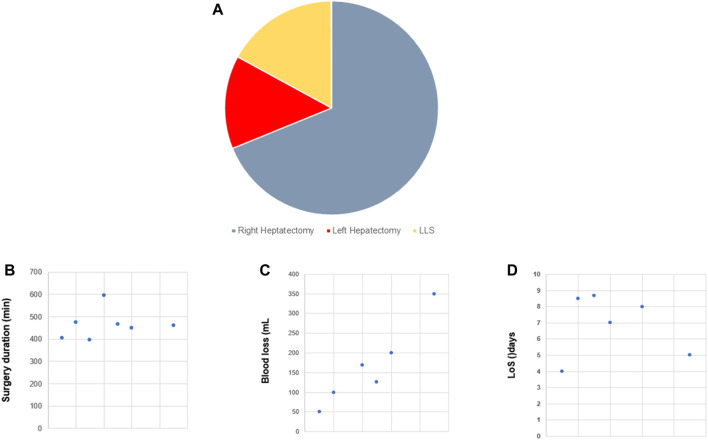
Robotic donor hepatectomy (R-DH). Overview of selected studies on R-DH (n = 13). **(A)** Pie chart illustrating the distribution between the different types of partial hepatectomy. **(B)** Dot plot of surgery duration [minutes]. **(C)** Dot plot of blood loss [mL]. **(D)** Dot plot of length of stay (LoS) [days].

Thirteen studies including 1629 patients undergoing robotic DH were reviewed [8, 14, 16–18, 20, 26, 41, 44, 70, 80, 87, 88]. Distribution of the types of partial hepatectomies is illustrated in Figure 3A, showing a majority of RH (69%). Conversion was indicated in 22/1629 (0.7%) patients (Table 1). Duration of surgery was typically between 400 and 500 min (Figure 3B), with blood loss essentially approximating 200 mL (Figure 3C). In term of postoperative outcomes, overall and major complications appeared in 145 (8.9%) and 38 (2.3%) patients, respectively. No case of postoperative mortality was reported (Table 1). LoS varied from 4 to 9 days (Figure 3D). In summary, R-DH appears as a safe procedure with low incidences of adverse events and no reported mortality, to date, given it is performed in centers with high expertise in MIS.

##### Patients’ Selection and Predictors of Adverse Outcomes

Like in L-DH, exclusion criteria essentially included high BMI, large graft volume or anatomical variants [14, 41, 44]. While predictors of adverse outcomes have been identified for L-DH, it precisely represents an unmet need in the field of R-DH. Future studies should actively tackle this challenge.

##### Comparison With Open and Laparoscopic Approaches

Studies compared R-DH with O-DH and/or with L-DH, tackling the stake question: does robotic offer any advantage in DH [8, 14, 16, 18, 20, 36, 40, 44, 46, 55]. Most comparisons showed that R-DH was associated with longer surgery duration, lower blood loss and similar postoperative complications rates [16, 18, 36, 40, 44]. Associations with lower pain (visual analogue scale on POD 3 of 2.4 in R-DH vs. 3.1 in O-DH, p < 0.001) [18] and shorter LoS (8 vs. 9 days, p < 0.001) [44] were also reported. The group of Riyadh recently published a landmark study providing a comprehensive analysis of 1724 donor-recipient pairs, and comparing 913 R-DH with 646 O-DH and 165 L-DH [20]. R-DH showed lower rate of overall complications (R-DH = 4%, L-DH = 8%, O-DH = 16%; p < 0.001) but major complications (R-DH = 0.1%, L-DH = 0%, O-DH = 0.8%; p = 0.065) and mortality (no case of mortality reported) were similar among the three groups. A study applying PSM to compare R-DH to L-DH, including 71 donor-recipient pairs in each group, reported reduced biliary after R-DH (22.5% *versus* 42.3%, p = 0.012) [16].

##### Outcomes After R-DH

Raptis DA et al. also analyzed outcomes of the recipients: both adult (R-DH = 23%, L-DH = 44%, O-DH = 31%; p = 0.001) and pediatric (R-DH = 15%, L-DH = 25%, O-DH = 19%; p = 0.033) recipients showed lower incidence of major complications after R-DH, as opposed to O-DH and L-DH. In 2024, the same group performed a fully robotic donor total hepatectomy and recipient liver graft implantation and therewith established an important milestone in the development of R-DH [25]. Likewise, propensity score matching was applied to compare 71 donor-recipients pairs undergoing either R-DH or L-DH, and specifically soughing biliary complications [16]. In donors, outcomes were similar but recipients of robotic-procured grafts showed lower rates of biliary complications (22.5% vs. 42.3%, p = 0.012), compared to recipients from L-DH. The authors attributed this difference to the precision of robotics for dissection and for bile duct division, which presumably reduced the risk of bile duct openings.

In a multicentric retrospective study using PSM, 50 recipients of robotic-procured grafts were compared to 100 recipients of open- and laparoscopic-procured grafts. Rate of major complications and survival were comparable among the groups [18]; another study by Amma et al. including 102 R-DH and 152 O-DH showed consistent findings [44].

##### Technical Considerations

Analysis suggested that 17 procedures were required to achieve the learning curve for robotic right donor hepatectomy [17]. Descriptions of surgical techniques are quite concordant among the different reports, at least for living donor right hepatectomy. DaVinci® system was the most used platform and surgeons typically placed 5 trocars. Most groups used a Pfannenstiel incision to extract the graft [8, 14, 16, 17, 26, 35, 36, 40, 41, 44, 46, 54, 55, 70, 87, 88]. Variations included Pringle maneuver and the use of indocyanine green cholangiography. The former was inconstant, described in some reports (on for 15 min, off for 5 min) [41], but seemed to be avoided by a majority of teams while it does not appear deleterious when applied [8, 17, 44]. Regarding the latter, it has been integrated in some surgical protocols to facilitate the visualization of the bile ducts before dividing them and thus presumably reduce the risk of biliary complications [8, 14, 16, 17, 36, 40, 41, 46, 54, 55, 87, 88].

Like in conventional liver surgery, parenchymal transection techniques and devices highly varied. Most studies described using harmonic scalpel and Maryland bipolar forceps [8, 14, 16, 17, 26, 36, 40, 41, 44, 46, 54, 55, 70], whereas a combined laparoscopic Cavitron Ultrasonic Suction Aspirator (CUSA) was also utilized in some cases requiring a second liver surgeon at the sterile operating table [26, 44]. Likewise, multiple techniques exist to divide bile ducts, but “clip and cut” was the most frequently reported option [14, 26].

### Minimally Invasive Liver Transplantation: Recipient’s Perspective

The first reported use of a minimally invasive recipient hepatectomy (MIRH) was in a Japanese study from Eguchi et al. with nine cases, mostly for viral chronic liver disease patients with a median Child-Pugh score of 9 [12]. Surgical technique consisted in a hand-assisted liver mobilization with a Gelport device inserted through an 8-cm upper midline laparotomy which was eventually extended to 12–15 cm to finish the explantation and perform the anastomoses. Median blood loss was 3940 mL and operative duration was 74min with one postoperative death. Results were not different from the 13 patients operated through a Mercedes-Benz-type incision during the same period, except for a longer median operative duration (812 vs. 741 min, p < 0.05).

The first report of a full laparoscopic explantation was published by Dokmak et al. in France in 2020 [13] in a patient with liver metastases of a neuroendocrine tumor. Without any underlying liver disease hence no portal hypertension and associated portosystemic shunts, portal flow must be preserved until the very end of the explantation. Rapid dissection of the bile duct and hepatic artery was performed with no porto-caval shunt, and extensive caval dissection was eased by the early division of the left hepatic vein trunk, aiming the shortest anhepatic phase duration. A previous 12-cm midline incision helped retrieve the specimen and perform a lateral clamping of the vena cava and anastomoses similar to the open approach. In this patient, a left lateral sectionectomy had to be performed. This report was later completed with a case series of 6 patients [43]. All patients had liver metastases from neuroendocrine tumors, all grafts were from brain death donors, midline incision length varied from 12 to 20 cm, blood loss from 250 to 600 mL, operative duration from 323 to 450 min and there was no postoperative death. Dokmak and colleagues emphasized the importance of small liver grafts of excellent quality, like in DH. Indications have been recently expended to selected cirrhotic patients with moderate liver volume and portosystemic venous shunts allowing early division of the portal vein with no portocaval anastomosis.

The first report of a full laparoscopic LDLT comes from Suh et al. in South Korea in 2021 [33]. The right liver graft from a living donor was inserted through a Pfannenstiel incision with laparoscopic implantation. Blood loss was 3300 mL, operative duration 960 min, warm ischemia time 84 min and portal clamping time 212 min. Left portal flow preservation technique was applied to shorten as much as possible the anhepatic phase. Laparoscopic anastomoses proved to be challenging, leading the same team to propose a hybrid approach, with robot-assisted arterial and biliary anastomoses, with blood loss of 11500 mL and operative duration of 1140 min [34]. In both cases there was no major complication and hospital stay were respectively 11 and 13 days.

In 2023, other pioneers pushed the envelope and published the first cases of fully robotic liver transplant, with R-DH followed by robotic graft implantation [25, 27]. Lee et al. reported blood loss of 6300 mL and operative duration of 850 min [27] while Broering et al. almost simultaneously reported a 3-case series with blood loss of 700–1000 mL and no major postoperative complication in both donors and recipients [25]. Eventually, Khan et al. performed a full robotic LT from a brain death donor with uneventful follow-up [85]. More recently, the groups from Lisbon and from Modena commonly reported their experience of robotic whole liver transplantation in 6 patients. Selection criteria were patients with hepatocellular carcinoma, small caudate lobe, low degree of portal hypertension, absence of porto-mesenteric thrombosis and low MELD score. Fully R-DH was followed by robotic implantation of the graft through a small midline incision. Reported outcomes were excellent: warm ischemia ranged from 55 to 90 min, surgery duration from 440 to 710 min. Altogether, 5/6 patients experienced no postoperative complication whereas one patient showed prolonged hyperbilirubinemia with no particular consequence [86].

Apart from these landmark publications, other reports were published between 2010 and 2025 representing a total of 35 patients (Supplementary Table S3) [21, 25, 30, 39, 47]. Procedures required five or six various size trocars, with pedicle dissection leaving long biliary and vascular stumps. Portal vein division was either performed during the pedicle dissection or at the latest point during the explantation (*i.e.*, left portal flow preserving dissection) [30, 33, 47]. Graft implantation was performed through a midline incision [12, 13, 21, 30, 43, 47] or a Pfannenstiel incision combined with a Gelport device [25, 27, 33, 34, 39]. Clamping of the inferior vena cava was lateral [13, 43], total with a Glover clamp (especially for minimally invasive implantation) [25] or with a combination of distal Chitwood clamp and proximal bulldog clamps [27, 39, 47]. In case of a right liver graft, iced gauze was put beneath the liver in the right upper abdominal quadrant [39, 47] and the graft portal vein was elongated during the backtable [39]. Minimally invasive anastomoses were robotic, hybrid or laparoscopic. Laparoscopy allows a larger range of movement and facilitates the presence of an assistant to position the iced gauze. Venous anastomoses are large enough to be performed laparoscopically [39, 47] whereas the robotic approach seems to be particularly adapted to the small diameter of the arterial and biliary anastomoses [27, 39, 47].

Throughout the literature, a total of 55 MIRH have already been performed. Operative time varied from 350 to 1065 min [13, 34], blood loss from 100 to 24200 mL [21, 30], intraoperative transfusion from 0 to 42 units of red blood cells [13, 30] and conversion rate from 0% to 60% during explantation [30]. Major complications (i.e., Clavien >2) occurred at most in 10% of patients [30]. Cold and warm ischemia times were not always reported but ranged respectively from 50 to 575 min and 21–117 min [30, 43, 47]. Operative and ischemia times as well as blood loss were greater in patients undergoing MIRH although postoperative outcomes did not seem to be worsened. This highlights the importance of the learning curve in such procedures, even considering that all surgeons involved are already highly skilled [39]. Coordination with the graft harvesting team is paramount to reduce ischemia time.

MIRH is feasible and challenges reside mostly in the implantation phase, where concerns can be raised about the necessity of vena cava total clamping, prolonged duration of the portal vein occlusion and its consequences especially in patients without portal hypertension. The hybrid laparoscopic/robotic approach seems to be a good alternative in the early experience with minimal risk for both recipients and grafts.

## Discussion

MILT is a rapidly emerging field, as exemplified by the rising number of publications during the last 5–10 years (Figure 1B). Tremendous progress has been made in a very short period of time as assessed by the number of publications and patients.

Obvious considerations and specificities render the use of minimal invasive techniques in LT much more complex which, given MILT controversial nature, limits its generalization. Conversely to conventional surgery that is typically performed in patients harboring diseases that indicate surgery, living donors are healthy by definition. Hence, safety becomes even more crucial in these patients. In addition, moderate or poor outcomes would likely discourage potential donors, which would ultimately accentuate the dramatic issue of organ shortage, particularly in Eastern countries where LDLT remains the main source of liver grafts. Therefore, most available studies previously discussed focused on safety. Recent studies provided valuable data that not only addressing safety or technical aspects of MILT, but aiming to identify risk factors or tackling the difficult challenge of patients’ selection. Improving patients’ selection is precisely at the crossroad between challenges and promises. It is likely a game-changer in MILT. It is a pivotal stake as important in donors as in recipients. For the latter, on a more technical point of view, patients’ selection must facilitate MILT procedures. Ideal recipients are those who need non complicated LT (e.g., no portal vein thrombosis) harboring small liver and small segment I, allowing easier manipulation and giving more space for instruments and cameras. Cirrhotic livers, stiff, are more difficult to retract and mobilize. Patients with ascites also were found to provide more workspace because of a dilated abdominal cavity. A left lateral sectionectomy can be performed to create space, minding a risk of disease dissemination in case of associated cancer disease. Presence of portal hypertension and collateral circulation can be beneficial by allowing rapid division of the portal vein without porto-caval shunt to ease caval dissection and increase tolerability of prolonged duration of portal and caval clamping. On the other hand, the absence of a porto-caval shunt increase mesenteric congestion and bleeding risk [25, 33, 43]. Along with the learning curve completion, indications are to be extended and future studies are needed to better understand how create the “bel-ensemble” and how pairing surgical approaches according to both donors’ and recipients’ characteristics. Presently, apart from feasibility, it is very early to conclude on the benefit of this approach regarding recovery, early and long-term complications.

Another challenge is the democratization of MILT. Although, certain groups have demonstrated the feasibility to start, develop and maintain MILT programs, achieving great results in short periods of time, it is a very demanding task. Again, MILT is essentially driven by a few groups, worldwide. In term of research, most articles provided data deriving from a single training cohort but lacked validation cohort. This is an important aspect that needs to be addressed by future studies in the field. Likewise, multicentric studies were quite uncommon.

A minimally invasive organ transplant consensus conference was held in Riyadh in December 2024. The aim was to develop consensus-driven recommendations for applying those techniques across various organ types (kidney, liver, pancreas, lung, heart, and uterus). The produced recommendations offer a guide for centers worldwide to implement MILT with ongoing evaluation and adaptation based on emerging evidence and technological advancements [89].

Drawing definitive conclusions about MILT from the literature is quite early. L-DH is the most studied field and the most performed procedure, with results backed by a sizable body of evidence. Recipient-related procedures are still confidential, with case reports or at best case series from highly-experienced surgeons. If one extrapolates the kinetics of MILT that occurred during the last 2-3 years, the field has a bright future. Promises rely on technological developments like the improvement of robotic platforms, for instance. The application of artificial intelligence is another important domain that has not yet been explored but that may offer pivotal options to overcome specific difficulties.

In summary, MILT is a rapidly emerging topic that gained a striking interest along the last years. Challenges and promises in MILT are closely related. Future studies may further tackle the challenge of patients’ selection and new technologies such as the application of artificial intelligence may be of interest to moving the field forward.
