# Correction: Achievements, Challenges and Promises of Minimally Invasive Liver Transplantation

**DOI:** 10.3389/ti.2026.16563

**Published:** 2026-03-25

**Authors:** Transplant International Production Office

**Affiliations:** Frontiers Media SA, Lausanne, Switzerland

**Keywords:** laparoscopy, minimally invasive surgery, minimally invasive liver transplantation, robotic surgery, liver transplantation

The artwork for Figures 2, 3 were placed in the wrong order. The artwork for figure two was incorrectly assigned to figure three. The artwork for figure three was incorrectly assigned to figure two. The order has now been corrected.

**FIGURE 2:**
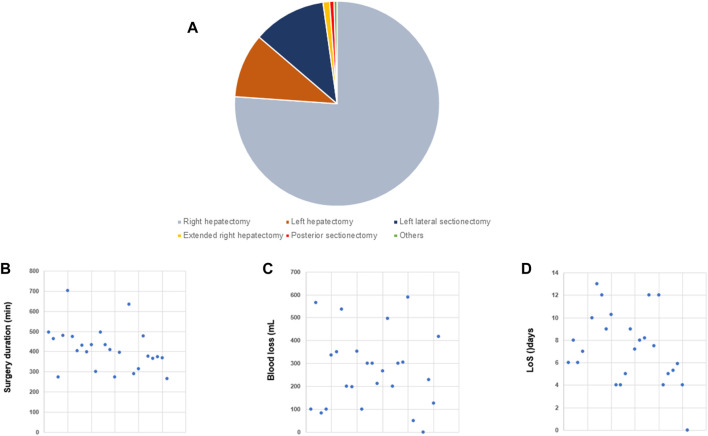
Laparoscopic donor hepatectomy (L-DH) Overview of selected studies on L-DH (n = 26). **(A)** Pie chart illustrating the distribution between the different types of partial hepatectomy. **(B)** Dot plot of surgery duration [minutes]. **(C)** Dot plot of blood loss [mL]. **(D)** Dot plot of length of stay (LoS) [days].

**FIGURE 3:**
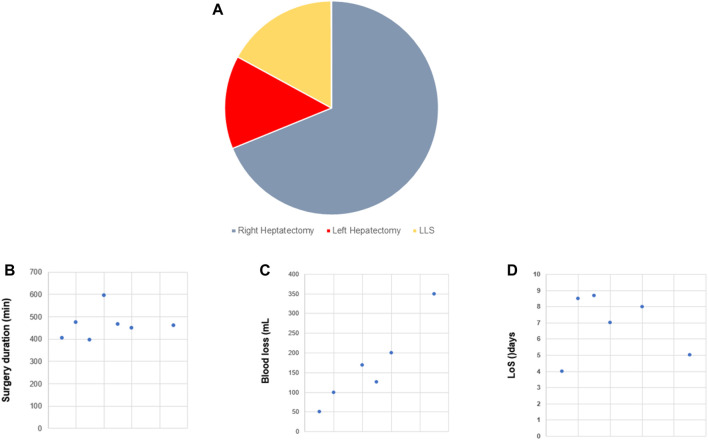
Robotic donor hepatectomy (R-DH). Overview of selected studies on R-DH (n = 13). **(A)** Pie chart illustrating the distribution between the different types of partial hepatectomy. **(B)** Dot plot of surgery duration [minutes]. **(C)** Dot plot of blood loss [mL]. **(D)** Dot plot of length of stay (LoS) [days].

The original version of this article has been updated.

