# Supplementary material for: Achievements, Challenges and Promises of Minimally Invasive Liver Transplantation
Source: Transpl Int. 2026 Feb 25;39:15366. doi: 10.3389/ti.2026.15366 (PMC12975614; doi:10.3389/ti.2026.15366)
Supplement: Supplementary file 1 [file Supplementaryfile1.docx]

**SUPPLEMENTARY MATERIALS**

**CAPSULE SENTENCE SUMMARY**

This article provides the synthesis of a comprehensive review of the available data on minimal invasive liver transplantation, covering both donor hepatectomy as well as graft implantation in recipients.

**SUPPLEMENTARY METHODS**

**Study selection**

The main criterion of inclusion was the use of minimally invasive surgical techniques in recipient and donor patients undergoing liver transplantation. Surgical approaches included laparoscopy, robotic and hybrid techniques. Both adult and pediatric liver transplantations were considered. Articles providing non-human data, such as *in vivo* or computerized models, were excluded.

Only original peer-reviewed articles were selected while reviews, editorials and meta-analyzes were excluded.

**Information sources and search**

Search was conducted in MEDLINE until March 1, 2025. Cross-referencing was also performed.

**Data charting process and items**

Data were extracted by CG and validated by IL and EK. Predetermined items included: (I) - Authors, (II) - Year of publication, (III) - City (Country), (IV) - Design and comparative analysis, (V) - Number of patients, (VI) - Type of surgery, (VII)- Duration of surgery, (VIII) - Blood Loss and Transfusion, (IX) - First warm ischemia, warm and cold ischemia, anhepatic phase, (X) – Conversion, (XI) - Overall and major complication, Post-hepatectomy liver failure, Primary non-function, Biliary leakage, Post-operative hemorrage , (XII)- Mortality, (XIII) – LoS, (XIV) - Techniques

**Synthesis of results**

Results were displayed in a narrative form and divided according to the subtypes of MILT.

Descriptive statistics were performed to provide a synthetic and quantitative overview of the available data. In case of multiple articles published by the same group, only the latest report was included in the analysis, to avoid duplicates. Continuous variables like duration of surgery, blood loss and LoS were inconstantly provided as mean or median values by the selected articles; for purpose of accuracy, those were thus illustratively displayed in dot plots.

Continuous variables were provided either as median [interquartile range] or mean [standard deviation] values, according to their distribution. Categorical variables were provided as frequencies with percentages. All statistics were performed with IBM SPSS Statistics, version 29.0.

**SUPPLEMENTARY TABLES**

**Supplementary Table 1: Summary of 54 articles about laparoscopic donor hepatectomy (L-DH)**

**Supplementary Table 2: Summary of 20 articles about robotic donor hepatectomy (R-DH)**

**Supplementary Table 3: Summary of 13 articles about MIS in recipients**
